# Crystal engineering of exemestane to obtain a co-crystal with enhanced urease inhibition activity

**DOI:** 10.1107/S2052252519016142

**Published:** 2020-01-01

**Authors:** Syeda Saima Fatima, Rajesh Kumar, M. Iqbal Choudhary, Sammer Yousuf

**Affiliations:** a H. E. J. Research Institute of Chemistry, International Centre for Chemical and Biological Sciences, University of Karachi, Karachi-75270, Karachi, Sindh 75270, Pakistan

**Keywords:** exemestane, anti-cancer compounds, thio­urea, crystal structure, Hirshfeld surface analysis, thermogravimetry, urease inhibition, crystal engineering, co-crystals, pharmaceutical solids

## Abstract

Co-crystallization is a phenomenon widely employed to enhance the physio-chemical and biological properties of active pharmaceutical ingredients (APIs). Exemestane, or 6-methyl­ideneandrosta-1,4-diene-3,17-dione, is an anabolic steroid used as an irreversible steroidal aromatase inhibitor, which is in clinical use to treat breast cancer. The present study deals with the synthesis of co-crystals of exemestane with thio­urea by liquid-assisted grinding. The purity and homogeneity of the exemestane–thio­urea (1:1) co-crystal were confirmed by single-crystal X-ray diffraction followed by thermal stability analysis on the basis of differential scanning calorimetry and thermogravimetric analysis. Detailed geometric analysis of the co-crystal demonstrated that a 1:1 co-crystal stoichiometry is sustained by N—H⋯O hydrogen bonding between the amine (NH_2_) groups of thio­urea and the carbonyl group of exemestane. The synthesized co-crystal exhibited potent urease inhibition activity *in vitro* (IC_50_ = 3.86 ± 0.31 µg ml^−1^) compared with the API (exemestane), which was found to be inactive, and the co-former (thio­urea) (IC_50_ = 21.0 ± 1.25 µg ml^−1^), which is also an established tested standard for urease inhibition assays *in vitro*. The promising results of the present study highlight the significance of co-crystallization as a crystal engineering tool to improve the efficacy of pharmaceutical ingredients. Furthermore, the role of various hydrogen bonds in the crystal stability is successfully analysed quantitatively using Hirshfeld surface analysis.

## Abstract 2

Co-crystals of the commercially available anticancer aromatase inhibitor exemestane (Ex) and guest co-former thio­urea were synthesized and the difference in the biological properties of the co-crystal compared with Ex was investigated. To study the various intermolecular interactions and their contribution to the structure stability, Hirshfeld surface analysis was successfully employed, and thermal stability was evaluated by DSC and TGA.

## 1. Introduction   

As defined by the FDA regulatory classification of pharmaceutical co-crystal guidance for industry, ‘co-crystals are crystalline materials composed of two or more different molecules, typically an active pharmaceutical ingredient (API) and a co-crystal former in the same crystal structure’. The approval of co-crystals by the FDA as new drug applicants has opened up new vistas in both the academic and the industrial research sectors (Gadade & Pekamwar, 2016 ▸), resulting in a significant increase in research in the field of co-crystals. It has already been established that the physical and biological properties of an API can be altered by co-crystallization (Caira *et al.*, 2012 ▸; Ghosh & Reddy, 2012 ▸; Issa *et al.*, 2012 ▸). The literature has disclosed many examples of fine tuning the biological and physical properties of many bioactive compounds and key pharmaceutical ingredients with suitable co-formers by using a number of crystal engineering approaches (Chen *et al.*, 2014 ▸; Charron *et al.*, 2013 ▸; Castro *et al.*, 2011 ▸). Examples include the co-crystallization of naturally occurring anti-leishmanial seselin (Hussain *et al.*, 2018 ▸), anti-tumour drug temozolomide (Sanphui *et al.*, 2013 ▸), antibiotic nalidixic acid (Gangavaram *et al.*, 2012 ▸) and well known quercetin (Smith *et al.*, 2011 ▸). Co-crystals are also known to have uses in thr agrochemical industry, as well as paint, electronic and optical materials (Blagden *et al.*, 2008 ▸; Papaefstathiou *et al.*, 2004 ▸; Sokolov *et al.*, 2006 ▸). The stabilization of co-crystals is known to be influenced by classical hydrogen bonding and π–π stacking. Non-covalent interactions (mainly hydrogen bonding) are involved in various biological systems due to their dynamic nature. The famous ‘lock-and-key’ model proposed by Emil Fischer (Fischer, 1894) for enzyme and substrate interactions has two main features: supramolecular chemistry and molecular recognition (Desiraju, 2001 ▸, 2000 ▸; Lehn, 1995 ▸). Therefore, it is worth studying both qualitatively and quantitatively the various interactions which contribute to crystal stability. Hirshfeld surface analysis (Spackman & Jayatilaka, 2009 ▸) is an effective approach for the qualitative and quantitative analysis of non-covalent interactions in crystal packing.

Enzyme inhibition is an important area of biomedical research contributing to the treatment of a wide range of disorders such as ulcers, cancer, inflammation, cardiovascular and central nervous system problems, and many infectious diseases. The binding of specific inhibitors to block the activity of an enzyme is the key to searching for potential drugs and to treating several physiological conditions associated with enzymes (Ramsay & Tipton, 2017 ▸). Urease is a pharmaceutically important and unique enzyme found in a wide variety of organisms and is known to catalyze the hydrolysis of urea into ammonia and carbonic acid (Amtul *et al.*, 2002 ▸; Mobley *et al.*, 1995 ▸). Urease also plays a key role in the formation of gastric ulcers (Mobley, 1996 ▸). The *Helicobacter pylori* infection (which can cause peptic ulcers and gastritis) is considered a worldwide problem with a high morbidity and mortality rate (Taha *et al.*, 2015 ▸). It is estimated that about 50% of the world’s population is infected with *H. pylori* (Rego *et al.*, 2018 ▸). The urease-producing pathogen (*H. pylori*) is reported to survive in the highly acidic environment of the stomach because the urease-catalyzed hydrolysis of urea produces ammonia, which forms a protective shield around the pathogen to protect it from the acidic conditions, and is the actual damaging factor for the host tissues, resulting in gastritis and gastro-duodenal ulcers (Rego *et al.*, 2018 ▸). Urea is a metabolic nitro­genous waste that is mainly produced in the liver, and the blood stream is responsible for carrying it towards the kidneys for excretion in urine. However, 20–25% of the urea remains in the body and is utilized as a substrate by the urolytic bacteria to produce high quantities of ammonia. During hepatic failure, the removal of toxic substances from the blood stream is affected and, as a result, the accumulation of toxic nitro­genous waste, including ammonia in the brain, is responsible for hepatic coma. The role of urease-producing pathogens is also reported in the progress of urinary catheter obstruction and in the production of kidney stones (urolithiasis) (Rego *et al.*, 2018 ▸; Maroney & Ciurli, 2014 ▸). Therefore, the search for possible inhibitors of this key enzyme is an important therapeutic need, as the currently available inhibitor drugs are reported to have many side effects (Kosikowska & Berlicki, 2011 ▸).

Exemestane (EX), or 6-methyl­ideneandrosta-1,4-diene-3,17-dione, is an anabolic steroid, used as an irreversible steroidal aromatase inhibitor. It is used clinically to treat breast cancer (Shiraki *et al.*, 2008 ▸). To the best of our knowledge, only one co-crystal of EX with maleic acid has been synthesized and reported by Shiraki and co-workers in 2008 in order to study the change in dissolution rate of poorly soluble APIs, *i.e.* EX.

By considering all of the above facts, the present study was conducted to observe changes in the biological activities of synthesized co-crystals of EX. The objective was to assimilate the co-former (thio­urea) in the molecular structure of the API (EX) to alter the crystal properties and therefore contribute to a change in the biological properties. Unfortunately, the synthesized exemestane:thio­urea (EX:TH) (1:1) co-crystal was found to be inactive for anti-cancer activity evaluated against the breast cancer cell line. The discouraging results regarding anti-cancer activity prompted us to evaluate the synthesized co-crystal against urease inhibition activity as our co-former (thio­urea) is already reported to be a tested standard in urease inhibition assays (Kanwal *et al.*, 2018 ▸). Synthesized co-crystals were found to be several folds more active than tested standard thio­urea. Therefore, as per the approved guidelines of the FDA, the synthesized co-crystal is a potential candidate as a urease inhibitor for further studies. The detailed structural features of the synthesized co-crystal were established on the basis of single-crystal X-ray diffraction (SCXRD) studies followed by qualitative and quantitative analyses of intermolecular interactions contributing to the co-crystal stability by Hirshfeld surface analysis. Differential scanning calorimetry (DSC) and thermogravimetric analysis (TGA) were also carried out to observe the thermal stability of the synthesized co-crystal.

## 2. Experimental   

### 2.1. Data collection and refinement   

Crystals of suitable sizes were mounted on a Bruker SMART APEX II X-ray diffractometer for data collection and structure determination. X-ray diffraction data were collected using Mo *K*α radiation (λ = 0.71073 Å). The program *SAINT* (Bruker, 2016 ▸) was used for data integration and reduction. Direct methods followed by Fourier transformation by employing full-matrix least-squares calculations were carried out to solve the structure by using *SHELXTL* and *SHELXL97* (Sheldrick, 1997 ▸) in the case of EX; the *APEX3* Suite combined with *SHELXL2014/7* and *SHELXL2016/6* (Sheldrick, 2015 ▸) was used in the case of the co-crystal. Intermolecular interactions were calculated using *PLATON* (Spek, 2009 ▸). The program *ORTEP3* (Farrugia, 1997 ▸) was utilized to generate a structural representation. *Mercury* 2.0 was used to create molecular graphics of the interactions (Macrae *et al.*, 2008 ▸). Crystallographic data, experimental details and structure-refinement parameters are summarized in Table 1 ▸.

### 2.2. Hirshfeld surface analysis   

Qualitative and quantitative analyses of various hydrogen bonds contributing to the co-crystal stability were carried out with the aid of Hirshfeld surface analysis, utilizing the software package *Crystal Explorer*. The two-dimensional Hirshfeld surface for the API is generated using *d*_norm_ parameters in the range −0.580–1.325, with a shape index of −0.996–0.994 and a curvedness of −4.029–0.281. Whereas for the co-former, the *d*_norm_ surface range is −0.612–1.059, the shape index is −0.998–0.996 and the curvedness is −4.000–0.40. The electrostatic potential surface has also been studied using the *TONTO* package incorporated in *Crystal Explorer* (Wolff *et al.*, 2012 ▸).

### 2.3. Synthesis and crystallization   

**Figure 1:**
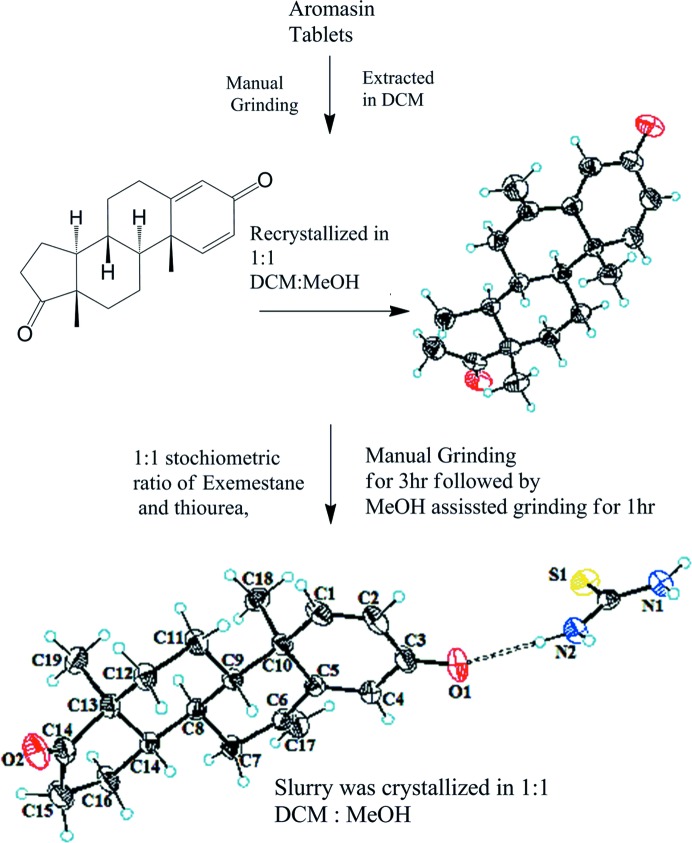
Schematic representation of the co-crystal synthesis.

Commercially available solvents and chemicals were utilized without any further purification. Thio­urea (TH) was purchased from Merck, Germany (index No. 612–082-0000). A small pestle and mortar was used for grinding. The commercially available drug Aromasin (1.411 g) was ground manually by using a pestle and mortar, and extracted in 100 ml di­chloro­methane (DCM) to obtain the API exemestane (0.350 g) after solvent evaporation under reduced pressure. The obtained API was further allowed to re-crystallize by dissolution in a 1:1 mixture of DCM and methanol after overnight evaporation. Manual grinding of crystallized EX (10 mg) and TH (20 mg) in a 1:1 stoichiometric ratio was carried out for 3 h, followed by methanol (1 ml) assisted grinding for 1 h. The resulting slurry was added to the DCM and methanol (1:1) and left overnight at room temperature (Fig. 1 ▸). The shiny co-crystals obtained (8 mg) were found to be suitable for SCXRD analysis.

### 2.4. Evaluation of biological activity   

Urease enzyme inhibition activity *in vitro* against the urease enzyme from Jack bean (*Canavalia ensiformis*, EC 3.5.1.5) was evaluated by adopting the same procedure as that described by Kanwal and co-workers (Kanwal *et al.*, 2018 ▸).

*In vitro* cytotoxicity and anti-cancer activity of the API and synthesized co-crystals were evaluated by using a standard MTT colorimetric assay as per the protocol adopted and explained by Park *et al.* (2008 ▸).

## 3. Result and discussions   

**Figure 2:**
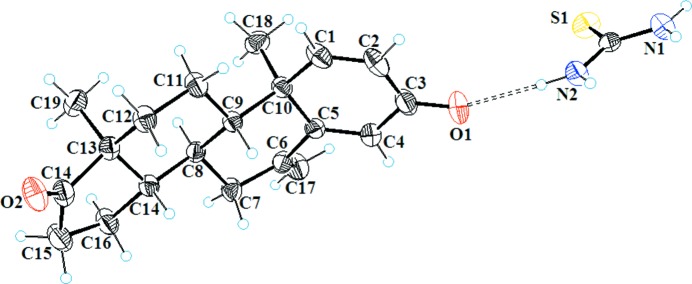
*ORTEP* drawing of the co-crystal of exemestane (6-methyl­ideneandrost-4-ene-3,17-dione) and thio­urea [(C_20_H_24_O_2_)(CH_4_N_2_S)] showing 30% probability ellipsoids.

**Figure 3:**
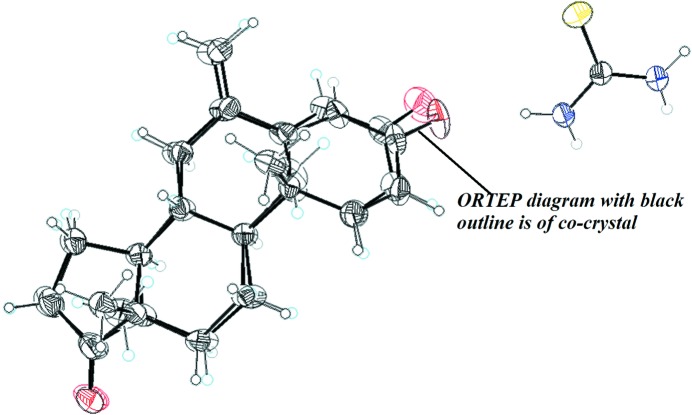
Overlapped *ORTEP* drawing of EX and the co-crystal with 30% probability ellipsoids.

SCXRD analysis revealed that EX crystallizes in the ortho­rhombic space group *P*2_1_2_1_2_1_ (Görlitzer *et al.*, 2006 ▸), whereas the co-crystal crystallizes in the monoclinic space group *P*2_1_; hence EX and TH are independent asymmetric moieties in the unit cell with *Z* = 2, as depicted in Table 1 ▸. Structural analysis revealed that EX and its co-crystal are both composed of four transfused rings (*A*–*D*). As a result of the C1=C2 and C4=C5 olefinic bonds in conjugation with the C3 carbonyl, ring *A* is planar in nature, with a maximum deviation of 0.05 (1) and 0.03 (3) Å from the root-mean-square plane (r.m.s.) for the C3 atom in EX and the co-crystal, respectively. Transfused rings *B* and *C* were found to exist in chair conformations, with puckering parameters (*Q* = 0.506 Å, θ = 169.13°, φ = 306.26°) and (*Q* = 0.549 Å, θ = 4.88°, φ = 142.90°), and (*Q* = 0.510 Å, θ = 12.40°, φ = 253°) and (*Q* = 0.546 Å, θ = 5.19°, φ = 276.96°) for EX and the co-crystal (Boeyens, 1978 ▸), respectively, whereas the five-membered ring *D* is folded like an envelope, with a maximum deviation of 0.262 and 0.265 Å for C14 with puckering parameters of *Q* = 0.4116 Å, φ = 206.59° and *Q* = 0.4124 Å, φ = 210.11° for EX and the co-former (Cremer & Pople, 1975 ▸). The α,β-unsaturated carbonyl carbon (C3=O1) was found to be involved in the strong conjugation with the C1=C2 and C4=C5 olefinic bonds. Two methyl groups were found to be axially oriented (Fig. 2 ▸). The conformational features of the EX molecule in the co-crystal were found to be similar to that of the individual molecule. In comparison, the difference between the bond length of the C3=O1 olefinic bond was found to deviate between 1.194 and 1.189 Å, with the orientation of the angle C2—C3—O1 being 121.85 and 122.54°, and C3—C4—O1 being 120.97 and 121.09° for EX and the co-crystal, respectively (Fig. 3 ▸).

### 3.1. Supramolecular features   

**Figure 4:**
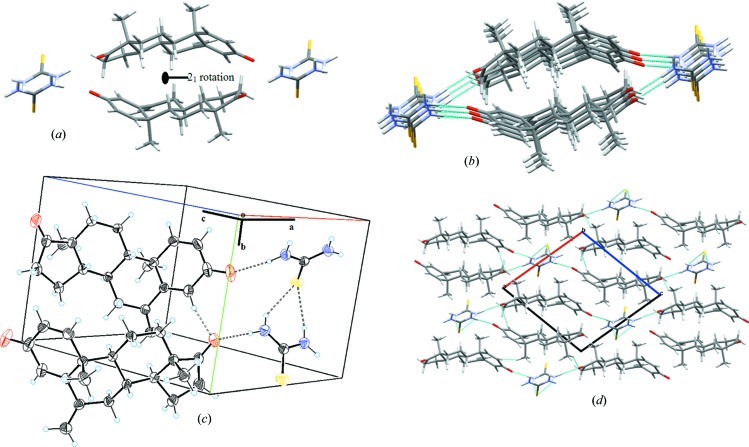
(*a*) 2_1_ axis rotation. (*b*) Parallel chain elongation along the *b* axis. (*c*) Unit cell packing diagram. (*d*) Intermolecular hydrogen bonding within the co-crystal, viewed along *b* axis to link neighbouring molecules to form a three-dimensional network.

Sulfur and nitro­gen atoms of TH play an important role in providing stability to the co-crystal via N(1)—H(1A)⋯S(1), N(1)—H(1B)⋯O(2), N(2)—H(2A)⋯S(1), N(2)—H(2B)⋯O(1), C(4)—H(4)⋯O(2) and C(20)—H(20A)⋯O(2) intermolecular interactions to form a three-dimensional network [Fig. 4 ▸(*c*)]. Among these interactions, C(2)—H(2B)⋯O(1) was found to be the strongest, with a bond length of 1.96 Å. N(1)—H(1A)⋯S(1) and N(2)—H(2A)⋯S(1) interactions result in the formation of an S6 ring graph set motif. N(1)—H(1B)⋯O(2) and N(2)—H(2B)⋯O(1) interactions are responsible for the two-dimensional unit cell packing; details of these interactions are summarized in Table 2 ▸.

**Figure 5:**
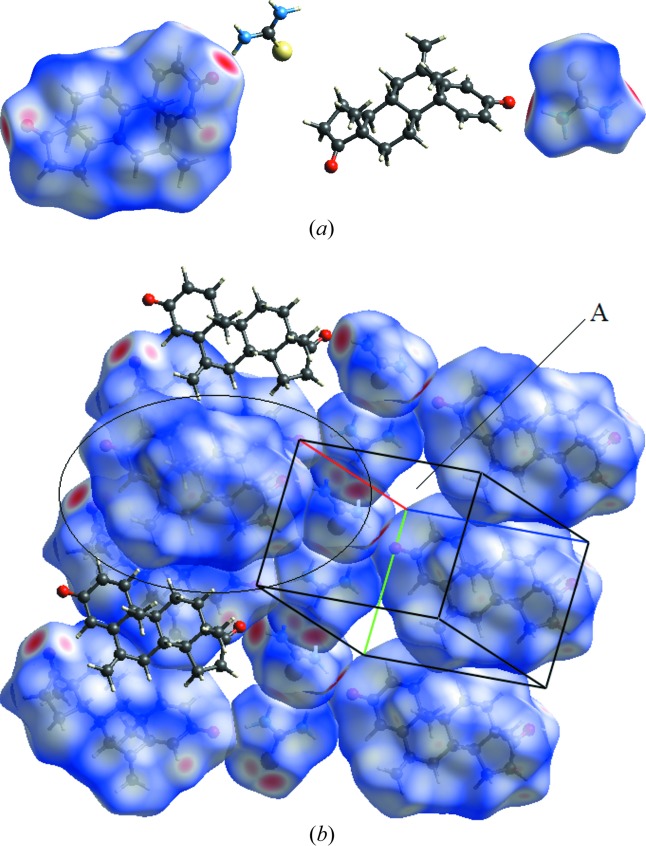
(*a*) Two dimensional Hirshfeld surface generated for the co-crystal. (*b*) Two dimensional Hirshfeld surface generated to show the compact nature of the Hirshfeld surface.

A two-dimensional Hirshfeld surface (Spackman & Jayatilaka, 2009 ▸) has been generated using *d*_norm_ [Fig. 5 ▸(*a*)]. In the Hirshfeld surface, the question of ‘compact packing’ arises (Spackman & Jayatilaka, 2009 ▸), as shown in the encircled area and empty *s* spaces (A) in the unit cell. This is answered by the expansion of crystal packing, revealing that the neighbouring molecule is encapsulating this gap [Fig. 5 ▸(*b*)]. The above situation is the main reason for analysing the API and co-former separately in qualitative and qualitative analyses, as they are involved in intermolecular interactions with neighbouring molecules.

**Figure 6:**
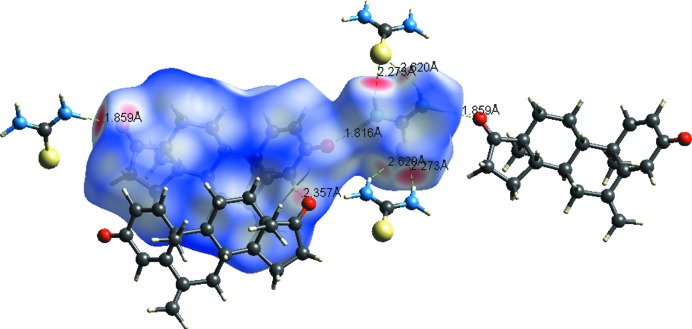
Intermolecular interactions with the two-dimensional Hirshfeld surface generated for the co-crystal.

Fig. 6 ▸ shows the intermolecular interactions of the API and co-former with the neighbouring moieties. S⋯H and the O⋯H are important interactions responsible for forming strong hydrogen bonds as indicated by the red spots on the Hirshfeld surface.

**Figure 7:**
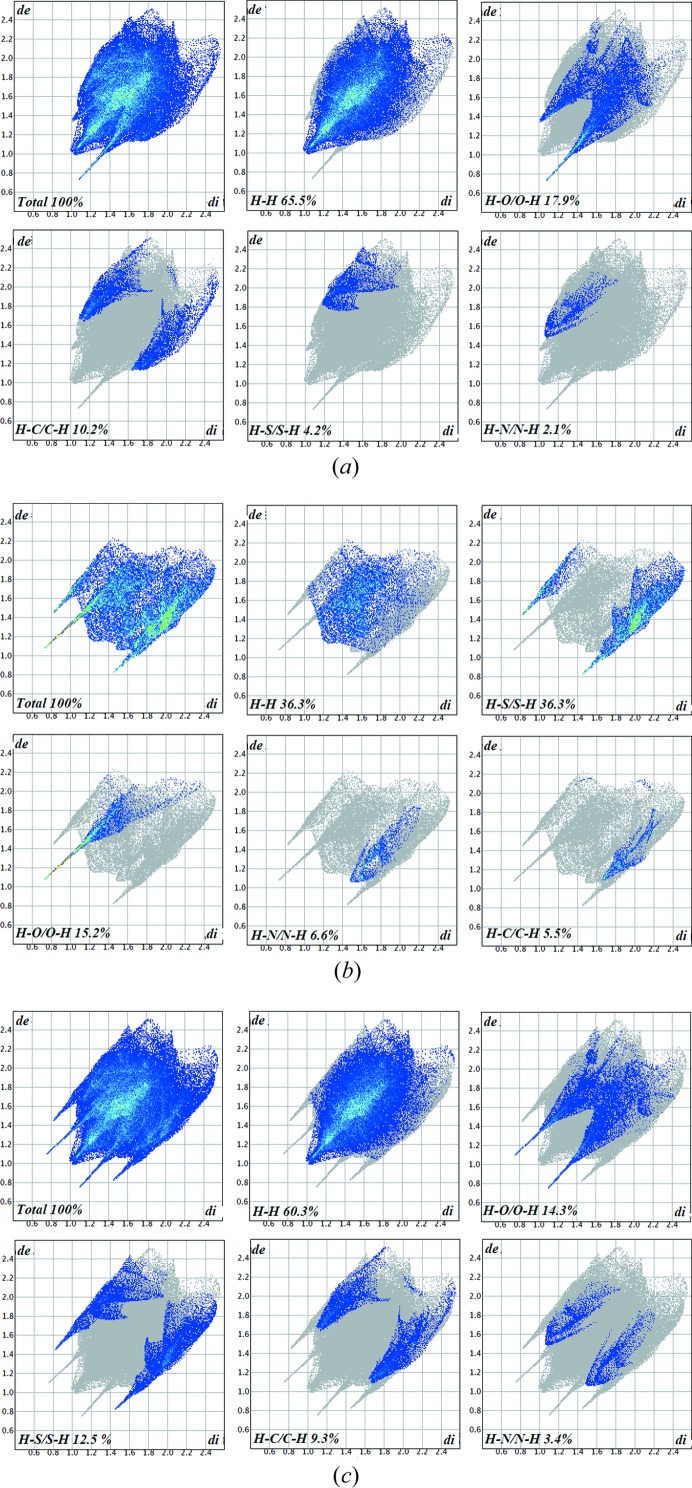
(*a*) Two-dimensional FPs of the API showing the percentage contribution of all contacts to the crystal packing. (*b*) Two-dimensional FPs for the TH moiety showing the percentage contribution of contacts to the crystal packing. (*c*) Two-dimensional FPs of the asymmetric unit.

**Figure 8:**
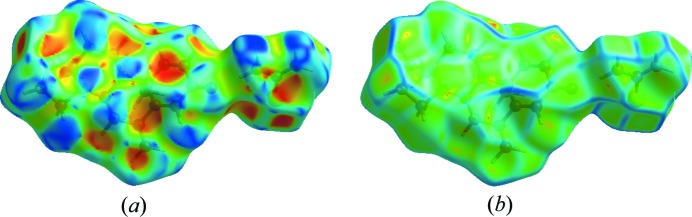
Shape index and curvedness mapped over the Hirshfeld surface.

**Figure 9:**
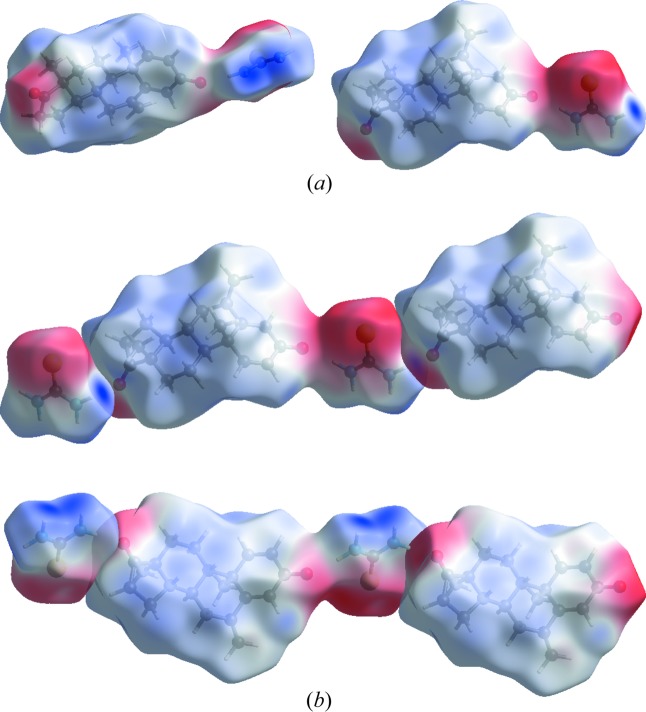
(*a*) Electrostatic potential surfaces mapped over the Hirshfeld surface to view the electropositive and electronegative sites. (*b*) Packing mode of the electrostatic potential surface.

Two-dimensional fingerprint plots (McKinnon *et al.*, 2007 ▸) (FPs) for the API [Fig. 7 ▸(*a*)] show the percentage contribution of contacts towards the crystal packing in which conventionally H⋯H contributes the most, amounting to 65.5%. O⋯H was found to be the strongest interaction among them all as shown by the sharp spike pointing to the origin and a 17.9% contribution to the unit-cell packing, followed by a 10.2% contribution from C⋯H. S⋯H and N⋯H contribute 4.2 and 2.1%, respectively; usually FPs generate the percentage contribution as a reciprocal for both distances *d*_e_ and *d*_i_. Here in the case of S⋯H and N⋯H for the API, cyan dots are only in the region of *d*e, which means S and N atoms are present only outside of the generated surface. In the case of the co-former [Fig. 7 ▸(*b*)], the size of the molecule is small, which is why the percentage contribution of H⋯H is equal to S⋯H, *i.e.* a 36.3% contribution to the unit cell packing. The O⋯H contribution is 15.2%, followed by contributions of 6.6 and 5.5% for N⋯H and C⋯H. In Fig. 7 ▸(*c*), FPs for the asymmetric unit have been drawn, which reveals that H⋯H contacts are at a maximum of 60.3%, whereas O⋯H and S⋯H are 14.2 and 12.5%, respectively; in addition to these contacts, C⋯H and N⋯H show contribution of 9.3 and 3.4% to the unit cell packing. Two valuable tools of the Hirshfeld surface, namely the shape index and curvedness, reveal information about the π–π stacking and the probability of forming interactions with neighbouring molecules, respectively. The shape index [Fig. 8 ▸(*a*)] surface showed blurred patches which reveal the presence of weak π–π stacking of the co-former moiety with neighbouring molecules. Curvedness [Fig. 8 ▸(*b*)] shows the packing probabilities at various positions of the surface generated, these bumps and hollows on the surface reflect the packing behaviour. The *ab initio* electrostatic potential (Fig. 9 ▸) (Spackman *et al.*, 2008 ▸) surface generated over the Hirshfeld surface shows the positive and negative potential sites of the molecule. In Fig. 9 ▸, blue dots over the surface relate the positions of the strong contacts to hydrogen-bond acceptors, whereas the red regions relate positions on the surface to hydrogen-bond donors (Fig. 9 ▸). The electrostatic potential map shows that the region of the surface where C=S of the TH moiety is more electronegative than the C=O attached to ring *D*.

### 3.2. Thermogravimetric analysis   

**Figure 10:**
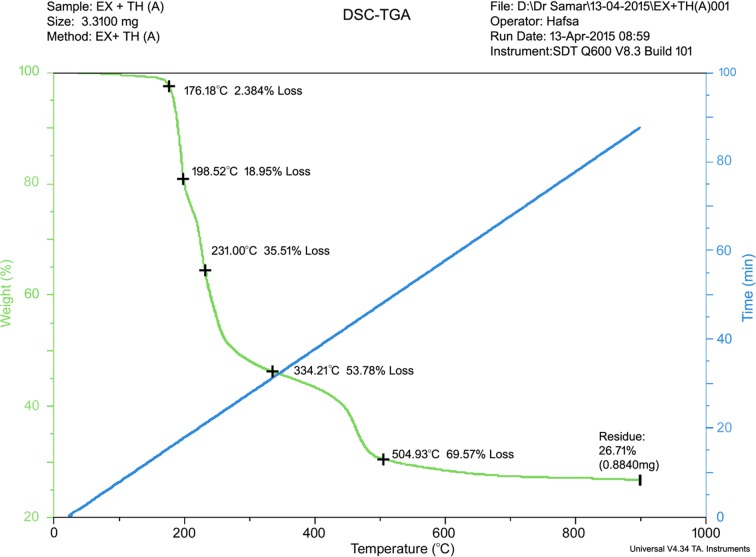
TGA Spectra of co-crystal (EX:TH).

**Figure 11:**
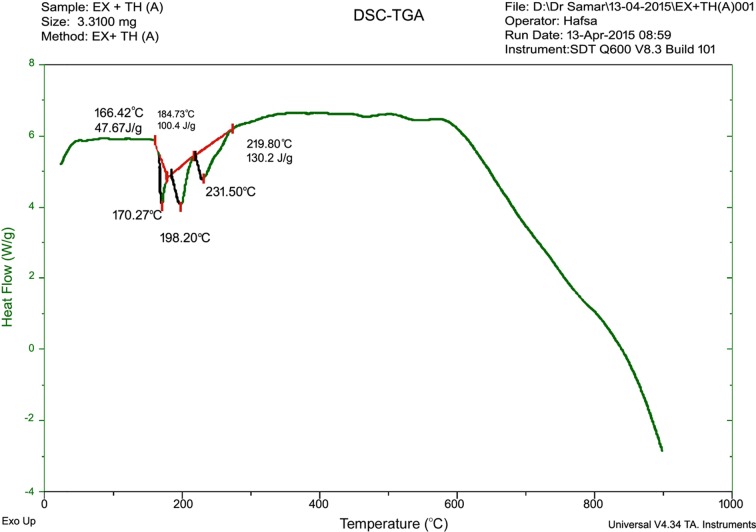
DSC Spectra of co-crystal (EX:TH).

From TGA studies (Steed, 2013 ▸) it is evident that the co-crystal (EX:TH) demonstrated a thermal stability up to 176.18°C, with a percentage weight loss of 2.384%. When the temperature was increased from 176.18 to 334.21°C, frequent percentage weight loss up to 51.396% was observed for the co-crystal (EX:TH) (Fig. 10 ▸). DSC spectra of the co-crystal (Fig. 11 ▸) clearly show three endothermic peaks observed at 170.27, 198.2 and 231.50°C due to the fusion of the TH crystal, thio­urea:exemestane co-crystal (EX:TH) and the degradation of the thio­urea polymer, respectively.

### 3.3. Biological activity   

EX has been reported as an anti-cancer agent against breast cancer, therefore, the synthesized co-crystal was first evaluated *in vitro* to observe any changes in its anti-cancer activity against the MCF-7 breast cancer cell line; however, it was found to be ineffective. On the other hand, the co-former (thio­urea) is reported as a tested standard to check urease inhibition activity *in vitro* (Kanwal *et al.*, 2018 ▸). Therefore, both the API (EX) and the synthesized co-crystal (EX:TH) were evaluated for their urease enzyme inhibition activity (*in vitro*) and interesting results were obtained. EX was found to be inactive against the urease enzyme. However, its co-crystal showed potent urease inhibition activity (IC_50_ = 3.86 ± 0.31µ*M*) and was found to be several-fold more active than standard TH (IC_50_ = 21.0 ± 1.45µ*M*). These promising results clearly indicate that the significant change in activity can be attributed to the synergistic effects of both the API and co-former; however, further studies are required to be able to comment on that. Results of the biological activity evaluation are summarized in Table 3 ▸.

## 4. Conclusions   

A co-crystal of the commercially available anti-cancer drug exemestane with thio­urea has been successfully synthesized with enhanced urease inhibition activity (*in vitro*) compared with that of the API (EX) and co-former (thio­urea) individually. The promising results of urease inhibition activity clearly demonstrate the possibility of the EX:TH (1:1) co-crystal as a new potential lead against peptic ulcers and uroli­thia­sis. The contribution of non-covalent interactions to the structural stability of the co-crystal was also evaluated quantitatively with the aid of Hirshfeld surface analysis, and clearly demonstrated the role of the amine and carbonyl functionalities of the co-former and API in forming various contacts in the crystal structure. Electrostatic potential analysis clearly identified the sites of the molecule that can be targeted for structural modification. The promising results of the present study clearly demonstrate the role of co-crystallization as an effective tool in the search for potential leads against various health disorders.

## Supplementary Material

Crystal structure: contains datablock(s) Exemestane, Exemestane-thiourea. DOI: 10.1107/S2052252519016142/ed5021sup1.cif

Selected geometric parameters for the exemestane-thiourea co-crystal. DOI: 10.1107/S2052252519016142/ed5021sup2.pdf

CCDC references: 1970916, 1970917

## Figures and Tables

**Table 1:** Experimental details

	Exemestane	Co-crystal
Empirical formula	C_20_H_24_O_2_	C_21_H_28_N_2_O_2_S
Formula weight	296.39	372.51
Temperature (K)	273 (2)	293 (2)
Wavelength (Å)	0.71073	0.71073
Crystal system	Orthorhombic	Monoclinic
Space group	*P*2_1_2_1_2_1_	*P*2_1_
Unit-cell dimensions (Å, °)	*a* = 9.959 (4), α = 90	*a* = 10.3797 (14), α = 90
	*b* = 11.731 (4), β = 90	*b* = 8.3706 (11), β = 105.568 (3)
	*c* = 12.842 (5), γ = 90	*c* = 10.7275 (14), γ = 90
Volume (Å^3^)	1500.2 (10)	897.9 (2)
*Z*	4	2
Density (calculated) (Mg m^−3^)	1.312	1.378
Absorption coefficient (mm^−1^)	0.083	0.199
*F*(000)	640	400
Crystal size	0.33 × 0.32 × 0.12	0.17 × 0.15 × 0.07
θ range (°)	2.352–28.365	1.971–28.390
Index ranges	−13 ≤ *h* ≤ 13, −15 ≤ *k* ≤ 14, −17 ≤ *l* ≤ 15	−13 ≤ *h* ≤ 13, −6 ≤ *k* ≤ 11, −14 ≤ *l* ≤ 14
Reflections collected	10477	6563
Independent reflections	3736 [*R*(int) = 0.0693]	3315
Completeness to theta	100.0%	25.242°
Refinement method	Full-matrix least-squares on *F* ^2^	Full-matrix least-squares on *F* ^2^
Data/restraints/parameters	3736/0/203	3315/1/244
Goodness-of-fit on *F* ^2^	0.968	1.034
Final *R* indices [*I* > 2σ(*I*)]	*R*1 = 0.0551, *wR*2 = 0.1005	*R*1 = 0.0472, *wR*2 = 0.0926
*R* indices (all data)	*R*1 = 0.1516, *wR*2 = 0.1329	*R*1 = 0.0733, *wR*2 = 0.1048
Absolute structure parameter	1 (3)	0.03 (14)
Extinction coefficient	0.012 (2)	NA
Largest difference, peak and hole (eÅ^−3^)	0.121 and −0.116	0.173 and −0.199

Computer programs: *SAINT*, *XPREP* (Bruker, 2016 ▸), *SADABS* (Sheldrick, 2014 ▸), *SHELXTL* (Sheldrick, 2008 ▸), *SHELXL2016/6* (Sheldrick, 2015 ▸).

**Table 2:** Hydrogen bonds for the co-crystal (Å and °)

*D*–H⋯*A*	*d*(*D*–H)	*d*(H⋯*A*)	*d*(*D*⋯*A*)	<(*D*H*A*)
N(1)—H(1A)⋯S(1)^i^	0.86	2.75	3.511 (3)	148.9
N(1)—H(1B)⋯O(2)^ii^	0.86	2.01	2.867 (4)	176.6
N(2)—H(2A)⋯S(1)^i^	0.86	2.42	3.253 (3)	164.4
N(2)—H(2B)⋯O(1)	0.86	1.96	2.821 (4)	174.2
C(4)—H(4)⋯O(2)^iii^	0.93	2.49	3.336 (5)	151.3

Symmetry transformations used to generate equivalent atoms: (i) −*x* + 1, *y* − 1/2, −*z*; (ii) *x* + 1, *y*, *z* − 1; (iii) −*x*, *y* + 1/2, −*z* + 1.

**Table 3:** Bioactivity of exemestane and the co-crystal

Sample	Urease inhibition activity (IC_50_ in µ*M* ± SEM)	Anti-cancer activity MCF-7 cell line (IC_50_ in µ*M* ± SEM)	Cytotoxicity against 3T3 normal fibroblast cell line (IC_50_ in µ*M* ± SEM)
EX (API)	NA	Not evaluated	>30
Co-crystal (EX:TH)	3.86 ± 0.31	Inactive	>30
Standard	Thio­urea 21.0 ± 1.45	Doxorubicin 0.924 ± 0.01	Cyclo­heximide 0.26 ± 0.12
