# Supplementary material for: Crystal engineering of exemestane to obtain a co-crystal with enhanced urease inhibition activity
Source: IUCrJ. 2020 Jan 1;7(Pt 1):105–12. doi: 10.1107/S2052252519016142 (PMC6949591; doi:10.1107/S2052252519016142)
Supplement: Supplementary file 2 [file m-07-00105-sup2.pdf]

IUCrJ

Volume 7 (2020)

Supporting information for article:

Crystal Engineering of Exemestane to Obtain Co-crystal with Enhanced Urease Inhibition activity

Saima Fatima Syeda, Rajesh Kumar, M. Iqbal Choudhary and Sammer Yousuf

S1. Selected geometric parameters (Å, º)

Table S1 Bond lengths [Å] and angles [°] for a.

S(1)-C(21)	1.626(4)
O(1)-C(3)	1.189(4)
O(2)-C(15)	1.161(4)
N(1)-C(21)	1.278(4)
N(1)-H(1A)	0.8600
N(1)-H(1B)	0.8600
N(2)-C(21)	1.282(4)
N(2)-H(2A)	0.8600
N(2)-H(2B)	0.8600
C(21)-N(1)-H(1A)	120.0
C(21)-N(1)-H(1B)	120.0
H(1A)-N(1)-H(1B)	120.0
C(21)-N(2)-H(2A)	120.0
C(21)-N(2)-H(2B)	120.0
H(2A)-N(2)-H(2B)	120.0
O(1)-C(3)-C(4)	121.1(5)
O(1)-C(3)-C(2)	122.5(5)
C(4)-C(3)-C(2)	116.4(3)
O(2)-C(15)-C(13)	125.9(4)
N(1)-C(21)-N(2)	116.6(3)
N(1)-C(21)-S(1)	122.2(3)
N(2)-C(21)-S(1)	121.2(3)

Torsion angles [°].

C(1)-C(2)-C(3)-O(1)	178.2(4)
O(1)-C(3)-C(4)-C(5)	-177.4(4)
C(12)-C(13)-C(15)-O(2)	29.6(6)
C(14)-C(13)-C(15)-O(2)	148.1(4)
C(20)-C(13)-C(15)-O(2)	-94.3(5)

Symmetry transformations used to generate equivalent atoms:
